# A 9-Step Theory- and Evidence-Based Postgraduate Medical Digital Education Development Model: Empirical Development and Validation

**DOI:** 10.2196/13004

**Published:** 2019-07-22

**Authors:** Robert de Leeuw, Fedde Scheele, Kieran Walsh, Michiel Westerman

**Affiliations:** 1 Athena Institute for Trans-Disciplinary Research VU University Amsterdam Amsterdam Netherlands; 2 British Medical Journal Learning British Medical Association House London United Kingdom; 3 Department of Internal Medicine Franciscus Gasthuis & Vlietland Hospital Rotterdam Netherlands

**Keywords:** postgraduate medical e-learning, instructional design, e-learning, distance education, design model, education, medical, education, distance, models, educational

## Abstract

**Background:**

Digital education tools (e-learning, technology-enhanced learning) can be defined as any educational intervention that is electronically mediated. Decveloping and applying such tools and interventions for postgraduate medical professionals who work and learn after graduation can be called postgraduate medical digital education (PGMDE), which is increasingly being used and evaluated. However, evaluation has focused mainly on reaching the learning goals and little on the design. Design models for digital education (instructional design models) help educators create a digital education curriculum, but none have been aimed at PGMDE. Studies show the need for efficient, motivating, useful, and satisfactory digital education.

**Objective:**

Our objective was (1) to create an empirical instructional design model for PGMDE founded in evidence and theory, with postgraduate medical professionals who work and learn after graduation as the target audience, and (2) to compare our model with existing models used to evaluate and create PGMDE.

**Methods:**

Previously we performed an integrative literature review, focus group discussions, and a Delphi procedure to determine which building blocks for such a model would be relevant according to experts and users. This resulted in 37 relevant items. We then used those 37 items and arranged them into chronological steps. After we created the initial 9-step plan, we compared these steps with other models reported in the literature.

**Results:**

The final 9 steps were (1) describe who, why, what, (2) select educational strategies, (3) translate to the real world, (4) choose the technology, (5) complete the team, (6) plan the budget, (7) plan the timing and timeline, (8) implement the project, and (9) evaluate continuously. On comparing this 9-step model with other models, we found that no other was as complete, nor were any of the other models aimed at PGMDE.

**Conclusions:**

Our 9-step model is the first, to our knowledge, to be based on evidence and theory building blocks aimed at PGMDE. We have described a complete set of evidence-based steps, expanding a 3-domain model (motivate, learn, and apply) to an instructional design model that can help every educator in creating efficient, motivating, useful, and satisfactory PGMDE. Although certain steps are more robust and have a deeper theoretical background in current research (such as education), others (such as budget) have been barely touched upon and should be investigated more thoroughly in order that proper guidelines may also be provided for them.

## Introduction

### Background

Medical educators have the responsibility to promote learning and create interventions and innovations to effectively help students develop proficiency in a broad spectrum of competencies [1]. One way of achieving this is by using digital education instruments, sometimes called e-learning or technology-enhanced learning. Digital education instruments can be defined as any educational intervention that is electronically mediated [2]. Some of these digital education instruments are theoretically grounded and are evidence based [3,4]. Studies have shown that digital education tools are at least as effective as other methods of training in psychomotor and nontechnical skills [5] and that the benefits are unparalleled accessibility and no time or location restriction [6]. However, there has been no consensus about the added value of digital education [2]. We postulate that this is partly because the focus of most studies has been on the learning goal (whether the learner achieved the curriculum goals or not), whereas we believe that the scope of outcomes should be broadened [7]. Our recent review showed that, apart from effectiveness, 4 other important aspects are looked at in postgraduate medical digital education (PGMDE): efficiency, motivation, usefulness, and satisfaction [8]. It is obvious that digital education has to be effective as well: learners must achieve the learning goal. But when evaluating digital education, aspects apart from the learning goal should be taken into account.

The abovementioned evaluated aspects depend on the content, but also on the instructional design (ID). In 1974, Snelbecker introduced the term “instructional design” as a link between the science of how people learn and daily practice as a process for designing instruction based on empirical principles [9]. Kemp et al described ID as a systematic method to manage the instructional process effectively so as to ensure competent performance by students [10]. In 2002, Merrill provided a very useful overview of various ID theories and models, concluding that they all shared a series of first principles, although no one theory or model included all principles. Differences can be based on different theoretical insights or in the details following the first principles, depending on, for example, the target audience [11]. Several such models are available to help experts in their quest to create, implement, and evaluate a digital learning experience [12], but none to date has aimed at PGMDE. Most models have been directed mainly toward educators, using abstract terms and theories that might not be useful for content experts with little educational experience.

### Objective

Previous literature has suggested that aiming an educational intervention at a specific target audience is most effective [13]. In line with this, we postulated that ID models should also be targeted as specifically as possible. We aimed this study at postgraduate medical professionals who work and learn after graduation. Arguments for such a specific target audience can be that adults might have different learning goals, working professionals might have specific motivational needs, and medical graduates might have a unique combination of clinic work and learning by doing [14-16] With this study, we aimed at providing a stepwise ID model for anyone planning to create PGMDE, to help them cover all important steps based on theory and current evidence.

## Methods

Intervention mapping is a process for developing theory- and evidence-based health education programs [17]. Analogous to the method of this model, we used our previous work to determine quality indicators, describe a working model, and compare that model with other available ID models.

### Quality Indicators

To create a specific ID model, we started in 2016 with an integrative literature review to evaluate which indicators, determining quality in PGMDE, were already available [18]. We searched a series of databases (PubMed, Web of Knowledge, CINAHL, PsycINFO, and Education Resources Information Center) and reviewed 11,093 articles. Ultimately, we used 36 relevant articles to gather 72 specifications that we found to be important for PGMDE. We divided these specifications into 6 domains, based partly on the International Organization for Standardization standard ISO-19796 [18,19]. We called this the postgraduate medical e-learning model (postgraduate MED model). These domains were preparation, software design and system specifications, communication, content, assessment, and maintenance.

In 2017, we discussed these 72 specifications in a series of focus group discussions with the most important stakeholders: medical education experts, postgraduate users, and commercial digital education creators [20]. The aim was to select which items were most relevant and which items experts and users would add to the list. The template analysis of these interviews provided us with 6 domains (preparation, motivators, barriers, learning enhancers, learning discouragers, and real-world translators) and 57 items. These domains gave us important insight into the main principles of PGMDE. This led to 3 main themes: motivate, learn, and apply.

To determine an international consensus on the 57 items from the focus group discussion, we performed a Delphi study in 2018 [21], aimed at identifying an empirically founded set of quality indicators for PGMDE. We asked a group of 13 international medical digital education experts and 10 experienced postgraduate users to rate the 57 items, explain why they would include or exclude the items, and add new items. After the first round, the group did not reach consensus on 20 items and added 15. After 2 rounds, the Delphi study produced a list of 37 indicators that we thereafter used as the basis for an ID model. For more details about the consensus rounds, refer to the previously published Delhi study [21].

### The Working Model

The abovementioned studies provided us with 3 themes, 6 domains, and 37 indicators. We then used our previous experience with creating PGMDE (eg, in gynecological ultrasound [22]) to order the items chronologically. The aim was to order them in such a way that model developers can follow the steps of the model without having to go back and forth in the creation process too often. The decisions in step 1 should be reflected in step 2, not the other way around.

### Comparing the Model With Other Instructional Design Models

The working model had to have two further characteristics: it had to add value to existing models, and it had to be as complete as possible. To determine the added value and to find possible missing steps, we compared the working model with 7 other ID models. We chose these models because our earlier systematic review showed that only these had been used in the evaluation or description of PGMDE [8]. The models with which we compared the steps are Kern’s 6 steps of curriculum development; the 4-component instructional design model (4C/ID) cognitive load principle; the ADDIE model (analysis, design, development, implementation, and evaluation); Gagné’s 9 events of instruction; the ASSURE (analyze the learner, state objectives, select media and materials, use media and materials, require learner participation, and evaluate and revise) model by Heinrich and Molenda; Merrill’s principles of instruction; and the Kemp ID model.

## Results

### Summary of Stages and Steps

**Figure 1:**
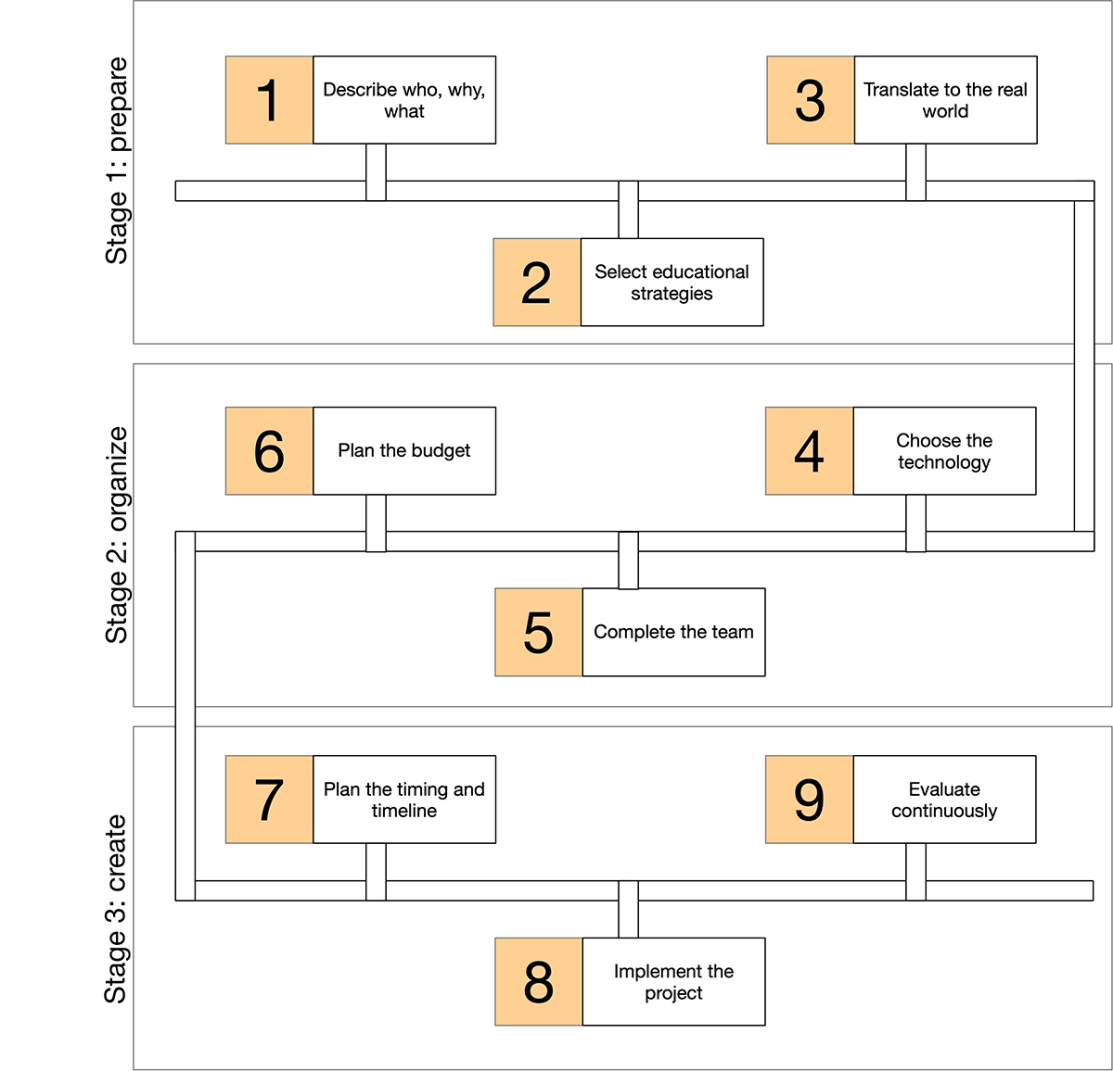
The postgraduate medical digital education model.

Three stages and 9 steps can be followed in chronological order to ensure that all 37 items are thought through and, when applicable, used for creating digital education interventions. Table 1 lists all the items from these previous studies, with the corresponding stages and steps. Stage 1 is *prepare*, stage 2 is *organize;* and stage 3 is *create*. We investigated each of these stages, explain the steps, and list the original items in each. Figure 1 summarizes all of the steps.

**Table 1:** The stages, steps, and principles of the postgraduate medical digital education model

Stages and steps		Principles
**Stage 1: Prepare**		
	Step 1. Describe who, why, what	1. Know your target audience
		2. Create a feeling of importance
		3. Convey a feeling of responsibility
		4. Take your user seriously
		5. Do not stress your user
		6. Do not force your user
		7. Define goals and objectives
		8. Inform the user about the goals and objectives
		9. Provide an overview of all lessons to be learned
	Step 2. Select educational strategies	10. Provide feedback
		11. Provide interactive elements
		12. Provide summaries
		13. Provide assessments
	Step 3. Translate to the real world	14. Provide real-world translation of the content
**Stage 2: Organize**		
	Step 4. Choose the technology	15. Ensure ease of navigation
		16. Design a clear layout
		17. Do not distract
		18. Make content adaptive
		19. Choose a flexible platform
		20. Make it easily accessible
		21. Make it safe and secure
		22. Have fast use and loading times
		23. Allow for nonlinear learning
		24. Personalize the learning path
		25. Show progress
		26. Select a learning environment
		27. Inform the user about optimal use
		28. Provide technical support
	Step 5. Complete the team	29. Add a content expert, medical educator, and information technology expert
		30. Prevent concern about the quality
		31. Identify the authors
		32. Provide references and sources
	Step 6. Plan the budget	33. Plan your budget
**Stage 3: Create**		
	Step 7. Plan the timing and timeline	34. Create a timeline
		35. Maintain
	Step 8. Implement the project	36. Update regularly
	Step 9. Evaluate continuously	37. Evaluate

### Stage 1: Prepare

One of many incentives may have pushed the creator to make the digital education intervention, for example, having been asked to do so by management, but it may also be the result of an internal motivation to share something or due to many other reasons. However, once the need is present, the first step should be to determine the goal of the digital education intervention, how it will educate, and its use for the learner. We called these domains motivate, learn, and apply.

#### Step 1: Describe Who, Why, What

The first step is to determine who, why, and what, which has a direct relation to motivating users. The *who*, or the target audience, must be defined as narrowly as possible. The more specific the definition, the better the content can be adapted. The first thing to realize is that the target audience is a digital learner who is not merely a consumer of technology, but who should realize the possibilities and potentials of digital technology and recognize the opportunity that it presents in their daily life [23]. Learner characteristics that can be used in the design should be taken into account, for example, online experience, age, cultural and social context, and educational culture [24]. It should, however, be kept in mind that the most important user factor is previous or existing knowledge, as this can then be properly built on [13].

When the target users have been identified, it is necessary to consider and communicate the *why*. This can be done by creating a feeling of importance for those users. When your users believe that undertaking the digital education intervention is important, they will be much more determined to do so. Attributing importance also helps to convey a feeling of responsibility not just for starting but also for completing the digital education intervention. These messages may be communicated when the digital education intervention is introduced or when people are invited to take part in it. Knowing your target audience will also help to prevent discouragement. Users can be discouraged by not being taken seriously (principle 4; eg, by childish syntax or drawings) or by being stressed (eg, by tight deadlines) or forced (eg, by being obliged to do something they consider not to be useful).

Creators must then carefully consider the *what*, that is, the goal and objectives of the digital education intervention (principle 7). Goals are broad or general and inform users about the aim of the whole curriculum or e-learning module. Objectives are specific and measurable and may include knowledge, skills, or attitudinal or behavioral goals [25]. When a clear goal and objectives have been set, it is crucial to inform users about them and provide an overview of all lessons to be learned. This should be done at the beginning of the digital education intervention, so the learner knows what to expect, but also during the digital education intervention to keep up with expectations.

#### Step 2: Select Educational Strategies

The second step is to consider *how* the targeted users will learn and which learning strategies are to be used. This depends greatly on the objectives defined in step 1, above. Instruments that may help in this process, as described in previous PGMDE studies, are problem-based learning [26], cognitive load theory [27], and multimedia learning [28]. Which strategy is the most effective for which goal will long remain a matter of debate; however, a guiding strategy must be chosen. According to previous studies, 4 instruments help creators facilitate efficient learning: feedback, interactive elements, summaries, and assessments.

#### Step 3: Translate to the Real World

The last step of the first stage is *apply*: translating the digital education intervention to the real world. Users want the digital education intervention to be useful. This can be achieved by different means, but the digital education intervention has to add something new to users that they can actually use in daily practice. This, therefore, concerns not only the learning goal and objectives but also the examples used in the digital education intervention. Questions to be considered are whether the feedback is written in a way that can be related to the users’ daily tasks and whether assessments not only serve an educational purpose but also give results that may be used when users return to work the next day.

### Stage 2: Organize

Completion of the first stage yields a good overview of the content of the digital education intervention: whom you target, what they should learn, how they can learn it best, and how the digital education intervention is to be kept as close as possible to the daily practice of the user. The next step entails organizing whatever is deemed necessary for the process of creating this digital education intervention. This may include the appropriate technology and a team to realize the plan; the financial recourses necessary must also be considered.

#### Step 4: Choose the Technology

When stage 1 is complete, the creator will have an idea of the technological needs, that is, how the technology should enable the previously set goals to be achieved. This is highly dependent on stage 1, but certain factors are universal. The aim of the technology should always be to achieve the stated curriculum goal by using the attributes of the supporting features. These are affordances (features that provide a potential for action), whereas constraints are those features that provide the structure of and guidance to those affordances [29]. Design elements must therefore always be borne in mind, such as ease of navigation and a layout that is clear, is not too distracting, and prevents nonadaptive content (content that does not change layout and design according to the device used). Decisions about the features should include consideration of a flexible platform that can be used on several devices and operating systems; be easily accessible, safe, and secure; have fast use and loading times; allow for nonlinear learning; personalize the learning path; and show progress. Finally, a learning environment must be selected and the user must be informed about the platform and the optimum device on which to access it, and technical support must be available.

#### Step 5: Complete the Team

Most digital education creators will probably already be working as part of a team. However, once a proper insight has been gained into the content and the technology needed, the team may be supplemented. It should contain at least one content expert, one medical educator, and one information technology expert. When the team is complete, its members must be asked to commit time and effort before the development is started. To prevent concern on the part of users about the quality of the digital education intervention, the identity of the authors should be clearly communicated alongside an explanation of their relevant expertise, and source information should be provided.

#### Step 6: Plan the Budget

To create any educational experience, a budget is necessary. This is determined by many factors. Little has been written about this and, to our knowledge, there is nothing specific for PGMDE. However, person-hours, materials, licensing, and technology are important topics to consider, and designers, editors, marketing, maintenance, evaluation, consultants, and overhead costs must also be borne in mind. It is estimated that 1 hour of digital education costs about 100 to 160 hours to create, with an average of US $18,750 in costs [30]. There are, however, ways to save on these costs, such as using free or low-cost recourses that already exist, making shorter courses that work on multiple devices, or using open source platforms and in-house faculty for the content [31].

### Stage 3: Create

When the above 2 important stages have been completed, creators will know what they want, what is necessary to achieve their aims, and who will help them. It is now necessary to plan the actual creation of the digital education intervention and start considering what will be necessary upon its completion. At this stage, a realistic timeline should be drawn up and planning for the implementation and evaluation should begin.

#### Step 7: Plan the Timing and Timeline

It will be necessary to plan and create a timeline for the creation of the digital education intervention to ensure the team meets that deadline. The timeline should not only be for the creation of the digital education intervention but should also be extended to consider its expiry date and the communication of that to the user, as well as the intervals at which the digital education intervention is to be maintained and updated. These are important subjects to consider at this stage: they might force a reconsideration of the budget, and communicating these dates and planned update logs to the learners is highly recommended.

#### Step 8: Implement the Project

The project can be implemented on several levels, but a minimum of 2 things must be determined. First to be determined is which factors are required for the digital education intervention to be implemented in the existing curriculum (eg, how the learners will be invited, whether management will offer support, whether any sort of marketing is necessary, or whether there will be a public introduction). Second to be determined is whether enough has been done to help learners implement their newly learned lessons in practice. (This has an overlap with real-world translation, but it is worth reconsidering how a user will actually use the digital education intervention.) This can be considered to be the same as other change management strategies or innovation implementation methods.

#### Step 9: Evaluate continuously

The final step is to evaluate (principle 37) and implement the plan. Our recent systematic review showed that PGMDE is mainly evaluated in terms of educational objective rather than design. In this review, only 4% of PGMDE studies used any form of evaluation of the curriculum design [8]. An evaluation strategy should be planned to answer the questions of what is desired, what must be evaluated, and what will be done with the resulting information, given that one part of the evaluation should be evaluating the implementation strategy itself.

### Comparing the Model With Other Instructional Design Models

Comparing the above 9 steps with the above-described other models, we found that the 9-step plan covered all the steps in other models, but that no other model covered all these steps. Table 2 overviews the steps in comparing the models. It shows how many items the models scored per step; Multimedia Appendix 1 shows which item was scored.

Kern’s 6 steps of curriculum development were described for the first time in 2002 and were aimed at curriculum developers responsible for the educational experience of students, residents, fellows, and faculty [25]. The 6 steps cover most of our 9-step model (see Table 1), but Kern’s program was not aimed at digital education. Therefore, there is little to no information on topics such as technology, budgets, updating, and the team required for digital education.

The 4C/ID model was initiated in 1992 and was aimed at prescribing how to develop educational programs that contain a mix of educational media, including text, images, speech, manipulative materials, and networked systems [27]. The 4C/ID cognitive load principle builds upon models of human memory and can be used to design training programs for complex learning. The focus of this model is therefore on learning aspects and how to make learning as efficient as possible. The model does not focus on any of the other domains.

The ADDIE model [32] was originally created to evaluate software and was first published in 1988 by Grafinger. As a more generic software development model, it relates closely to the 9-step model. The 5 steps of the ADDIE model can be split up into smaller steps, and the only thing left unconsidered by the ADDIE model is budget. Even though the design step considers educational strategies, the focus is much more on technology than learning and therefore misses domains such as budget and maintain.

Gagné’s 9 events of instruction were introduced in their first form in 1992. This is a very complete model for learning, taking into account several learning theories, the ADDIE model, Keller’s ARSC (attention, relevance, confidence, satisfaction) model, and evaluation instructions [33]. Although the ADDIE model refers to evaluation, the 9 events of Gagné do not. Neither does the Gagné model discuss implementation, updates, team, or budget.

**Table 2:** Comparison of instructional design models by score (number of steps covered).

Model	Stage 1: prepare	Stage 2: organize	Stage 3: create
9-step model	5/5	3/3	4/4
Kern	5/5	0/3	2/4
4C/ID^a^	3/5	0/3	0/3
ADDIE^b^	5/5	2/3	3/4
Gagné	5/5	1/3	0/4
ASSURE^c^	5/5	1/3	2/4
Merrill	2/5	1/3	0/4
Kemp	4/5	1/3	1/4

^a^4C/ID: 4-component instructional design model.

^b^ADDIE: analysis, design, development, implementation, and evaluation.

^c^ASSURE: analyze the learner, state objectives, select media and materials, use media and materials, require learner participation, and evaluate and revise.

The ASSURE model was developed by Heinich and colleagues in 1999 and “is an instructional model for planning a lesson and the technology that will enhance it” [12,34]. It consists of 6 steps aiming to produce more effective learning and teaching. Although the design step does consider technology, it is not aimed at digital education, with all its technological challenges. Steps such as budget, timeline, and team are not included in the ASSURE model.

The first principles of instruction by Merrill is a series of 5 principles common to various theories aiming to promote learning [11], published in 2002. The 5 principles focus on the learning domain almost exclusively, although technology can be considered to be covered by the demonstration principle. Domains as learning goals and educational strategies are not mentioned.

The Kemp ID model from 2007 is the result of several disciplines in ID [10]. It is distinguished by its circular approach, allowing for continuous evaluation of all steps, which is more dynamic and fluid than the linear approach taken by other models. Although it covers behavioral and cognitive approaches, it does not cover real-world translation or technology-related domains such as budget, team, timeline, updates, or implementation.

## Discussion

### Principal Findings

The postgraduate MED model is, to our knowledge, the first ID model for PGMDE. Compared with other models, it is unique in two ways. First, it is based on 37 building blocks, which are evidence-based items based on 3 empirical studies and on the collaboration of experts and experienced users. While most other models are combinations of theories and expert opinion, the 9-step model presented here combines theory with published reports, expert opinions, and consensus. Second, it is the only model that covers a wide range of steps aimed directly at digital education and postgraduate education. It can be debated whether such a model may also be used for other kinds of target audiences. We aimed to make the stages and steps broad, but the 37 indicators we used are quite specific. Whether these indicators are also applicable to other audiences, which might be missing, such as graduates, has not yet been investigated. The broad subjects of this model, on the other hand, make it very suitable for content experts with little experience in creating a curriculum. Educators may find many of the steps to be obvious. Even so, the aim was to stimulate debate within the development team about each step. There might not be an optimal educational strategy for each scenario, but the use of cognitive load theory and multimedia learning theory seems useful in daily practice [27,35,36]. We believe that the benefit of these models is not only in the sound theory behind them, but also that they are specific enough to provide easy-to-follow instructional principles. Following these principles, the 4 mentioned instruments appear promising: feedback, interactive elements, summaries, and assessments. According to the cognitive load theory, learning occurs when the information is chunked, which is done in the feedback, assessment, and summaries. Another way is repetition, which can also be found in feedback, summaries, and assessments. According to cognitive load theory, using the information actively helps to move the information into long-term memory. This is done by using feedback, interactive elements, and assessments. Therefore, these instruments not only seem to have been effective in published reports [37,38], but are also grounded in theory.

Another promising aspect of digital education is adaptive learning environments. Unique to digital learning is that each individual can have an experience based on her or his own needs and desires, a form of individual learning without the time and costs of one-on-one human tutoring. Digital learning allows a more intelligent system to interpret the learner’s previous use. It can then adapt content, nonlinear learning paths, multimedia, and tools to a personalized learning experience. Studies have shown an increasing interest in the added value of adaptive learning environments [39,40].

Other reviews have shown the added value of creating a curriculum with the help of learning and designing models [7]. It is clear that the planning of an educational experience is far from simply adding some online presentations and that the lack of ID leads to unanticipated and unexplained learning outcomes. Educational theory can be used to create the ID to develop effective, appealing, consistent, and reliable instruction [41]. The structure of a model like this also helps to identify those points that are efficient and those that require improvement [42].

### Limitations

The biggest limitation of the postgraduate MED model will be the ways in which an educator can interpret each step. A model like this implies that a curriculum may be designed by simply following a few steps. However, the whole is much more complex, and each step is worth a great deal of thought, consideration, and awareness of other theories and models. Much can be said to focus a model on a specific part of a learning experience, for example, pedagogic theory. Yet we wanted to provide an overview so that educators might realize how complex digital learning is and should be. This model may be considered different from other models perhaps because the people making those other models wanted an in-depth focus on a certain subject, rather than trying to create an all-in-one solution. We do not believe this 9-step model is such a solution, although danger lies in oversimplification.

### Further Research

Having an overview of these 9 steps reveals the gaps in the literature. While many theories and studies have been performed on the effectiveness of learning [1], almost nothing is known of other subjects. Our insight into the budgets needed or expected to create digital education interventions has rarely been described. More should be written on the experience of others, for example, the number of hours taken, the main costs, and the personnel or team chosen to limit these. Little is also known of the ways to properly evaluate the design. Most models tell users to evaluate, but there are no validated evaluation instruments that look at the design. The same is true for implementation. We should consider how to implement the digital education intervention into the working life of the learners, but little is known of how to do that and what may be used as outcomes for successful implementation. Implementation of digital education has an analog with implementing innovations. There are models for the implementation of innovations, such as Kotter’s 8-step model [43] and Rogers’ model of diffusion of innovation [44]. To our knowledge, these models have not been used for the implementation of digital education, but it seems a very interesting future research path.

### Conclusion

We have described a complete set of evidence-based steps, expanding a 3-domain model (motivate, learn, and apply) into an ID model that can help every educator in creating efficient, motivating, useful, and satisfactory PGMDE. The postgraduate MED model is underpinned by aspects derived from other dominant models and should provide enough basics to start the journey of creating digital education. Much remains to be learned, and the next most logical step would be to validate an evaluation instrument of the digital education design.

## Appendix

Multimedia Appendix 1 Comparison of design models.

## Abbreviations

4C/ID: 4-component instructional design model
ADDIE: analysis, design, development, implementation, and evaluation
ARSC: attention, relevance, confidence, satisfaction
ASSURE: analyze the learner, state objectives, select media and materials, use media and materials, require learner participation, and evaluate and revise
ID: instructional design
postgraduate MED model: postgraduate medical e-learning model
PGMDE: postgraduate medical digital education
