# Supplementary material for: A 9-Step Theory- and Evidence-Based Postgraduate Medical Digital Education Development Model: Empirical Development and Validation
Source: JMIR Med Educ. 2019 Jul 22;5(2):e13004. doi: 10.2196/13004 (PMC6876560; doi:10.2196/13004)
Supplement: Multimedia Appendix 1 [file mededu_v5i2e13004_app1.docx]

| **Eight-step model**  Appendix A 1 – comparing design models | **Kern** | **4C/ID** | **ADDIE** | **Gagne** | **ASSURE** | **Merrill** | **Kemp** |
| --- | --- | --- | --- | --- | --- | --- | --- |
| Target audience | Targets learners |  | Analysis | Recall prior knowledge | Analyse learners | Use existing knowledge | Identify characteristics learners |
| Why | Needs assessment |  | Analysis | Present content material | Prepare learner participation |  | Clarify course content |
| Goals and objectives | Goals and objectives | Educational goals | Design | Orient the learner | State objectives |  | Define objectives |
| Educational strategy | Educational strategies | Supporting information | Design | Gain attention | Select strategy |  | Structure content, design instructional strategy |
| Real World translation | Problem identification and general needs assessment | Procedural information and task support | Implement | Enhance retention and transfer |  | Real world problem, apply and integrate in learner’s world |  |
| Technology |  |  | Development | Provide learner guidance, elicit practice, feedback, assess | Select technology, media and material, utilise technology | Demonstrate | Plan appropriate model of delivery, choose appropriate resources |
| Team |  |  | Analyse |  |  |  |  |
| Budget |  |  |  |  |  |  |  |
| Timeline |  |  | Analysis |  |  |  |  |
| Update And maintain |  |  |  |  | Revise |  |  |
| Implement | Implementation |  | Implement |  |  |  |  |
| Evaluate | Evaluation |  | Evaluation |  | Evaluate |  | Develop evaluation instrument |
